# Supplementary material for: Microglia Reduce Herpes Simplex Virus 1 Lethality of Mice with Decreased T Cell and Interferon Responses in Brains
Source: Int J Mol Sci. 2021 Nov 18;22(22):12457. doi: 10.3390/ijms222212457 (PMC8624831; doi:10.3390/ijms222212457)
Supplement: Supplementary file 1 [file ijms-22-12457-s001.zip › ijms-1452605-supplementary.pdf]

Table S1. Primer sequences for RT-PCR

Genes	Forward Primer (5′-3′)	Reverse primer (5′-3′)
IFN-β	GGTGGAATGAGACTATTGTTG	AGGACATCTCCCACGTC
IFN-γ	TTCTTCAGCAACAGCAAGGC	TCAGCAGCGACTCCTTTTCC
CXCL10	GCTGGGATTCACCTCAAGAA	CTTGGGGACACCTTTTAGCA
Mx1	CTGAGATGACCCAGCACCTGAA	CTCCAGGAACCAGCTGCACTTAC
IL-1β	GCAAGTGTCTGAAGCAGCTATG	CCACAGCCACAATGAGTGATAC
IL-6	CCTCTGGTCTTCTGGAGTACC	ACTCCTTCTGTGACTCCAGC
iNOS	CAGCTGGGCTGTACAAACCTT	CATTGGAAGTGAAGCGTTTCG
TNF-α	ATGAGCACAGAAAGCATGA	AGTAGACAGAAGAGCGTGGT
Arginase-1	CTCCAAGCCAAAGTCCTTAGAG	AGGAGCTGTCATTAGGGACATC
IL-10	ATAACTGCACCCACTTCCCA	GGGCATCACTTCTACCAGGT
TGF-β	CCTGCAAGACCATCGACATG	TGTTGTACAAAGCGAGCACC
β-actin	AACCCTAAGGCCAACCGTGAAAAGAT GACC	CCAGGGAGGAAGAGGATGCGGC
CD45	GAACATGCTGCCAATGGTTCT	TGTCCCACATGACTCCTTTCC
CD4	GTTCAGGACAGCGACTTCTGGA	GAAGGAGAACTCCGCTGACTCT
CD8	ACTACCAAGCCAGTGCTGCGAA	ATCACAGGCGAAGTCCAATCCG

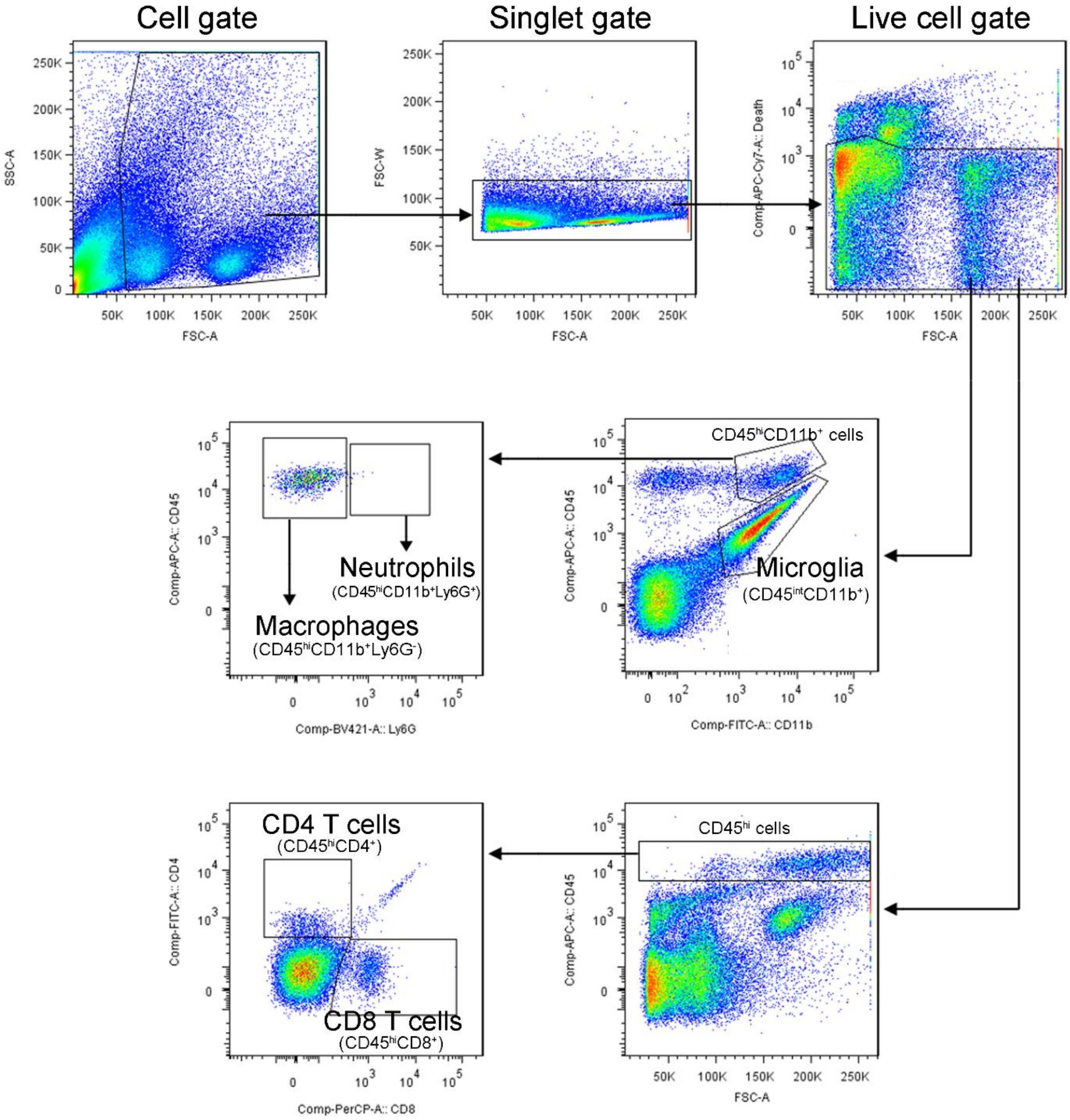

Figure S1. The gating strategies of flow cytometric assays. Immune cells isolated from the mouse brain were assayed for markers of microglia (CD45^{int}CD11b^{+}), macrophages (CD45^{hi}CD11b^{+}Ly6G^{-}), neutrophils (CD45^{hi}CD11b^{+}Ly6G^{+}), CD4 T cells (CD45^{hi}CD4^{+}), and CD8 T cells (CD45^{hi}CD8^{+}) by flow cytometry. The gating strategies to identify these cells are shown.

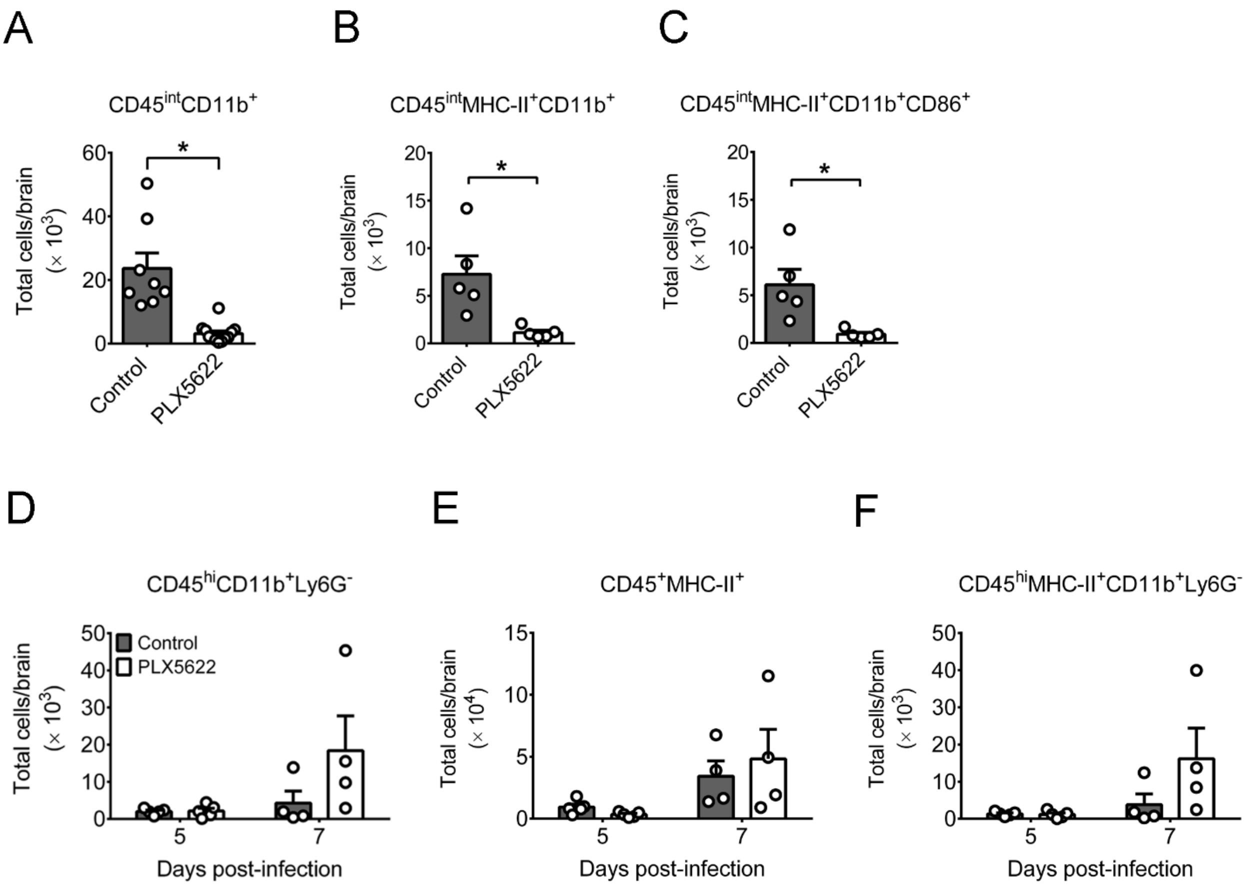

Figure S2. PLX5622 fails to reduce the infiltration of myeloid cells and activation of infiltrating myeloid cells in brains of infected mice. The brains of mice fed with control chow or the chow containing PLX5622 and infected for 5 days were assayed by flow cytometry for **(A)** microglia (CD45^{int}CD11b^{+}) and activated microglia by detecting the levels of activation markers, **(B)** MHC-II with anti-MHC-II antibody (clone I-A/I-E; BD Biosciences) and **(C)** CD86 with anti-CD86 antibody (clone GL-1; BioLegend). The brains of infected mice were harvested on indicated days p.i. and assayed for **(D)** macrophages (CD45^{hi}CD11b^{+}Ly6G^{-}), **(E)** activated leukocytes (CD45^{+}MHC-II^{+}), and **(F)** activated macrophages (CD45^{hi}MHC-II^{+}CD11b^{+}Ly6G^{-}). The data represent means + SEM (error bars) of 4-8 samples per group obtained from at least two independent experiments. *, $P < 0.05$, via a Mann-Whitney *U* test.

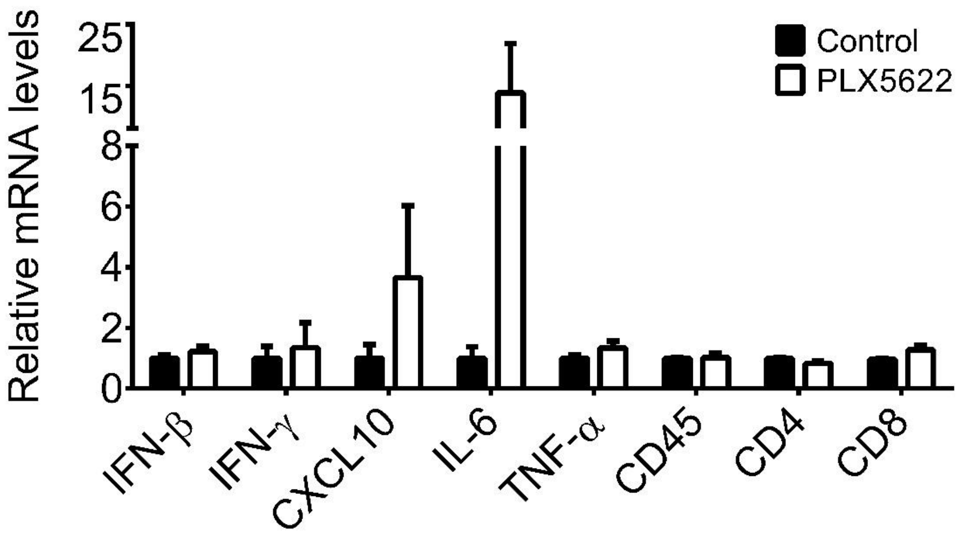

Figure S3. Effects of PLX5622 on the expression of immune responses in eyes of infected mice. Eyes of infected mice fed with control chow or the chow containing PLX5622 were harvested on day 5 post-infection, processed, and assayed by quantitative RT-PCR for mRNA levels of indicated genes. The data are expressed as the ratio of indicated mRNA normalized to β-actin mRNA in one eye, and the means of control groups were set as 1. The data represent means + SEM (error bars) of 3 samples per group.
